# Supplementary material for: Developing an Echocardiography-Based, Automatic Deep Learning Framework for the Differentiation of Increased Left Ventricular Wall Thickness Etiologies
Source: J Imaging. 2023 Feb 18;9(2):48. doi: 10.3390/jimaging9020048 (PMC9964852; doi:10.3390/jimaging9020048)
Supplement: Supplementary file 1 [file jimaging-09-00048-s001.zip › jimaging-2170776-supplementary.pdf]

Supplement

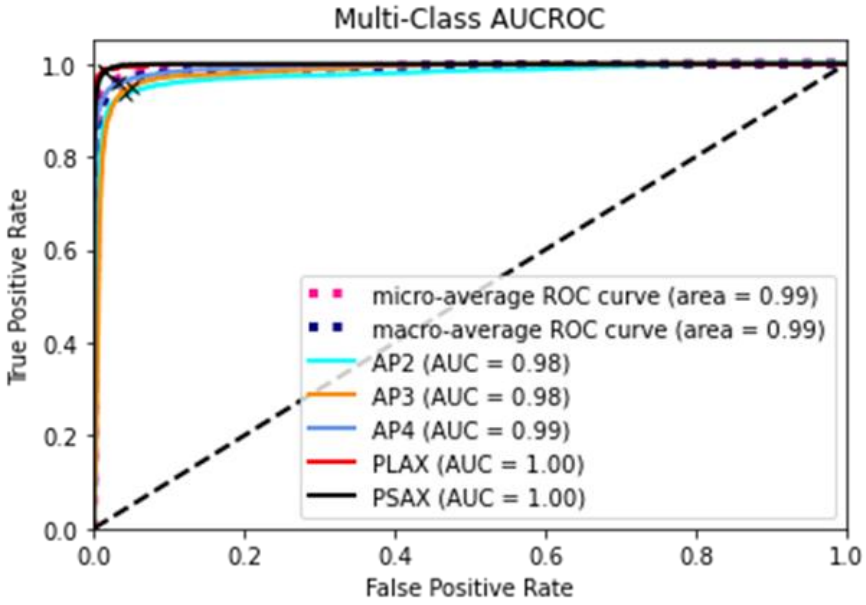

Figure S1. The receiver operating curves for the view classification.

Table S1. The Cohen Kappa coefficients of agreement between two readers

	**AI**	**Reader 1**	**Reader 2**	**Reader 3**
**AI**	1.000	0.755	0.792	0.750
**Reader 1**	--	1.000	0.800	0.794
**Reader 2**	--	--	1.000	0.812
**Reader 3**	--	--	--	1.000
